# Morphological aspects and distribution of granules composed of deproteinized bovine bone or human dentin into a putty mixture: an in vitro study

**DOI:** 10.1186/s13005-023-00398-7

**Published:** 2023-12-18

**Authors:** Inês Pimentel, Bruno Henriques, Filipe Silva, Oscar Carvalho, Wim Teughels, Mutlu Özcan, Júlio C. M. Souza

**Affiliations:** 1 Center for Microelectromechanical Systems (CMEMS), University of Minho, Guimarães, 4800-058 Portugal; 2 Associate laboratory (LABBELS), University of Minho, Guimarães, Braga, 4710-057 Portugal; 3 Ceramic and Composite Materials Research Group (CERMAT), Dept. of Mechanical Engineering (EMC), Federal University of Santa Catarina (UFSC), Florianópolis, SC 88040-900 Brazil; 4 Department of Oral Health Sciences, Periodontology, Dentistry, University Hospitals Leuven, Katholieke Universiteit Leuven, Leuven, 3000 Belgium; 5 Clinic for Masticatory Disorders and Dental Biomaterials, Center of Dental Medicine, University of Zurich, Zurich, 8032 Switzerland; 6 Center for Interdisciplinary Research in Health (CIIS), Faculty of Dental Medicine (FMD), Universidade Católica Portuguesa (UCP), Viseu, 3504-505 Portugal; 7 University Institute of Health Sciences (IUCS), CESPU, Gandra PRD, 4585-116 Portugal

**Keywords:** Dentin graft, Deproteinized bovine bone, DBBM, Bone healing

## Abstract

**Objective:**

The main aim of this study was to evaluate the morphological aspects and distribution of granules composed of deproteinized bovine bone mineral (DBBM) and human dentin-derived bone graft (HDBG) into a putty consistency mixture.

**Materials and methods:**

DBBM or HDBG were mixed with an alginate-based hydrogel at two different granule/hydrogel ratio (1:1 and 1:3) and divided into four test groups while two control groups were composed of DBBM or HDBG free of hydrogel. Groups of specimens were cross-sectioned for morphological evaluation by scanning electron microscopy (SEM) at backscattered electrons mode. Details on the dimensions and pores’ size of DBBM and HDBG were evaluated after mixing different amounts of particles and alginate-based hydrogels.

**Results:**

Microscopic analyses revealed a size of DBBM granules ranging from 750 up to 1600 μm while HDBG particles showed particle size ranging from 375 up to 1500 μm. No statistical differences were identified regarding the size of granules (*p* > 0.5). The mean values of pores’ size of DBBM particles were noticed at around 400 μm while HDBG particles revealed micro-scale pores of around 1–3 μm promoted by the dentin tubules (*p* < 0.05). The lowest distance between particles was at 125 μm for HDBG and 250 μm for DBBM when the particle content was increased. On decreasing the particles’ content, the distance between particles was larger for DBBM (~ 1000 μm) and HDBG (~ 1100 μm). In fact, statistically significant differences were found when the content of granules increased (*p* < 0.05).

**Conclusions:**

The increased content of bioactive ceramic granules in a putty consistency mixture with hydrogel decreased the space among granules that can promote a high ceramic density and stimulate the bone growth over the healing process. Macro-scale pores on bovine bone mineral granules stimulate the formation of blood vessels and cell migration while the micro-scale pores of dentin-derived granules are proper for the adsorption of proteins and growth of osteogenic cells on the bone healing process.

**Clinical significance:**

A high amount of bioactive ceramic granules should be considered when mixing with hydrogels as a putty material since that result in small spaces among granules maintaining the bone volume over the bone healing process. Deproteinized bovine bone mineral granules have macro-scale pores providing an enhanced angiogenesis while dentin-derived granules possess only micro-scale pores for the adsorption of proteins and proliferation of osteogenic cells on the bone healing process. Further studies should evaluate the combination of different bioactive ceramic materials for enhanced bone healing.

## Introduction

After the loss of a tooth, the alveolar bone undergoes a continuous process of resorption [1]. Remodeling of bone tissue following tooth extraction or extensive craniomaxillofacial defects results in bone loss that can negatively affect the rehabilitation with endosseous implants. Adequate bone height and width are essential for further placement of endosseous implants [1, 2]. Guided bone repair (GBR) is a surgical technique using bone grafting materials to support bone healing in cases of tissue damage. Inorganic bone substitutes have advantages in the formation of nucleation sites for cell adhesion and bone formation [2, 3]. Nowadays, several bone substitutes are used to improve bone healing and the materials can be classified according to their origin as autogenous, xenogenous, allogeneic, and alloplastic [1, 4–7]. Autogenous bone graft (autograft) material is considered the first-choice biologic material due to their osteoinductivity and osteoconductivity, as well as providing osteogenic cells and bone growth factors [5]. Autografts confer a lower risk of immunological rejection and a strong healing mechanism within proliferation of cells, angiogenesis, and bone growth. Still, they have disadvantages such as the limited amount of graft material available, additional surgical site, donor site morbidity, and extra surgical expenses [1, 4, 8].

Deproteinized bovine bone mineral (DBBM) is the most widely used xenograft in dentistry since the success in bone healing is well-reported in literature. The inorganic bone network of DBBM reveal three-dimensional morphological features similar to that found in human cancellous bone. The removal of organic components is achieved through chemical and thermal treatments that preserve the trabecular joint and porosity of the bone tissue composed mainly of hydroxyapatite (Hap). Thus, DBBM acts as an osteoconductive material, with a porosity of around 75%, providing the ideal environment for angiogenesis and new bone formation [6, 9, 10]. Block or granules of DBBM have been used in surgical procedures that require an enhanced ridge contour or volume stability of the damaged site. The extracted tooth of patients has also been considered an attractive source of bone substitute since it consists of an autogenous material without a secondary harvesting surgery [1, 6, 11, 12]. The dentin tissue is composed of approximately 70% Hap, 20% organic materials, and 10% water that depends on the tooth region. The organic matrix is composed of collagen fibers and proteins such as growth factors and bone morphogenic proteins (BMP) [13–16]. The demineralization process of the dentin graft maintains the bioavailability of non-collagenous proteins that increase osteoinductivity. After demineralization, dentin tubules become channels which adsorb and release proteins which can support the adhesion, proliferation, and differentiation of osteogenic cells [8]. Then, the success of bone healing also depends on the capability to oxygenate, supply nutrients, and removing waste products from the graft material.

Several studies have shown that particle size strongly influences osteoconduction and new bone density [6, 10, 13]. Thus, a highly porous three-dimensional network allows mesenchymal cells to infiltrate, attach, proliferate, and differentiate [6, 7, 12]. Besides physicochemical nature, strength, and biocompatibility of bone substitutes, the investigation of morphological aspects is crucial for correlation with enhanced bioactivity and the bone healing process [17, 18]. In this way, in vitro studies are required to evaluate the morphological aspects and distribution of particulate bone graft prior to the surgical application.

Thus, the purpose of this study was to inspect the morphological aspects and distribution of human dentin-derived bone graft (HDBG) or deproteinized bovine bone mineral (DBBM) particles on mixing with alginate-based hydrogels. It is hypothesized that the dimensions, content, and distribtuion of DBBM and HDBG granules vary into a putty consistency mixture regarding the clinical handling, volume, and morphological aspects of the particulate materials.

## Materials and methods

### Preparation of the specimens

Deproteinized bovine bone mineral (DBBM) was assessed in this study as provided by the manufacturer (Biograft™, Ossmed, Cantanhede, Portugal). Briefly, DBBM fragments were industrially produced using a chemical treatment followed by thermal treatment up to 1200^o^ C to remove the organic compounds and maintaining the hydroxyapatite network. Then, DBBM was milled in a high-efficiency ball mill (90s; 8000 M Mixer, SPEX, Metuchen, NJ, USA) and then in planetary ball mill (350 rpm, 5 min; PM 100, Retsch, Germany). Granules were selected using gradual mesh resulting in DBBM granules with size ranging from 300 up to 1200 μm in diameter. The chemical composition (%wt) of the DBBM granules given by the manufacturer was the following: 32 C, 9.2 O, 8.1 Ca, 3.3P. The ratio of Ca/P was at 2.45.

On harvesting human dentin-derived bone graft (HDBG), extracted third molars from human donors were firstly immersed in distilled water for 10 min and then in a solution of 2% sodium hypochlorite (NaOCl) for 10 min. Afterwards, teeth were immersed in 10% formalin solution for 7 days. Finally, teeth were stored in 0.9% NaCl solution for hydration over a period of 7 days prior to the milling procedure. Each tooth was stored in separate sterilized crystal containers at room temperature, labelling each container with the characteristics of the teeth (type, weight, dimensions). Diamond burs were used to remove remnant periodontal ligament, tissue, and debris from the tooth surfaces. Teeth roots and enamel were removed to harvest the dentin tissue. Then, teeth were immediately milled with the Smart Dentin Grinder™ apparatus (KometaBio Inc., Cresskill, NJ, USA). The milling process resulted in dentin particles (granules) ranging from 300 up to 1200 μm. The dental particles were then immersed in an isopropyl alcohol solution in a sterile container for 10 min to dissolve all organic debris and bacteria. Then, dentin granules were placed in ethylenediaminetetraacetic acid (EDTA) for 2 min for partial demineralization and then washed in sterile saline solution for 3 min. Thus, microorganisms were eradicated after the cleaning procedure due to a strong alkali combination between NaOH and ethanol.

All procedures performed involving teeth from human donors followed the ethical standards of the research committee of the University Institute of Health Sciences (IUCS, Portugal) and therefore with the 1964 Helsinki declaration and its later amendments or comparable ethical Standards (Ethical Protocols Number 13/CE-IUCS/2022). The project was previously reviewed and approved by an institutional review board. Each participant was in good oral health, with no history of antibiotic treatment during the previous 6 months.

**Fig. 1:**
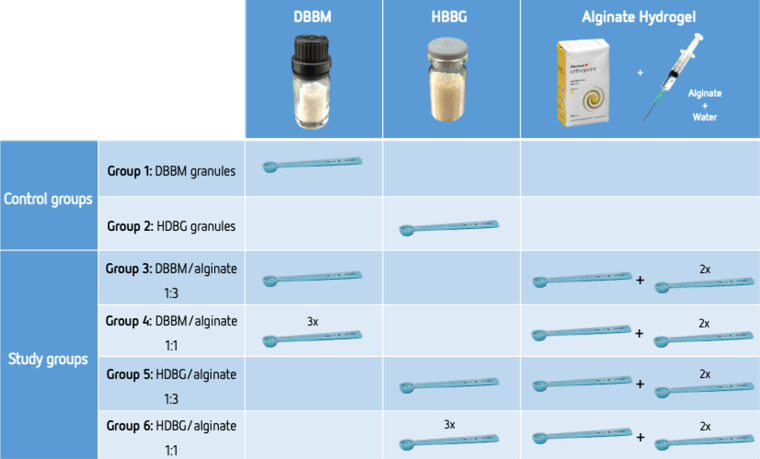
Schematics of the groups of materials

**Fig. 2:**
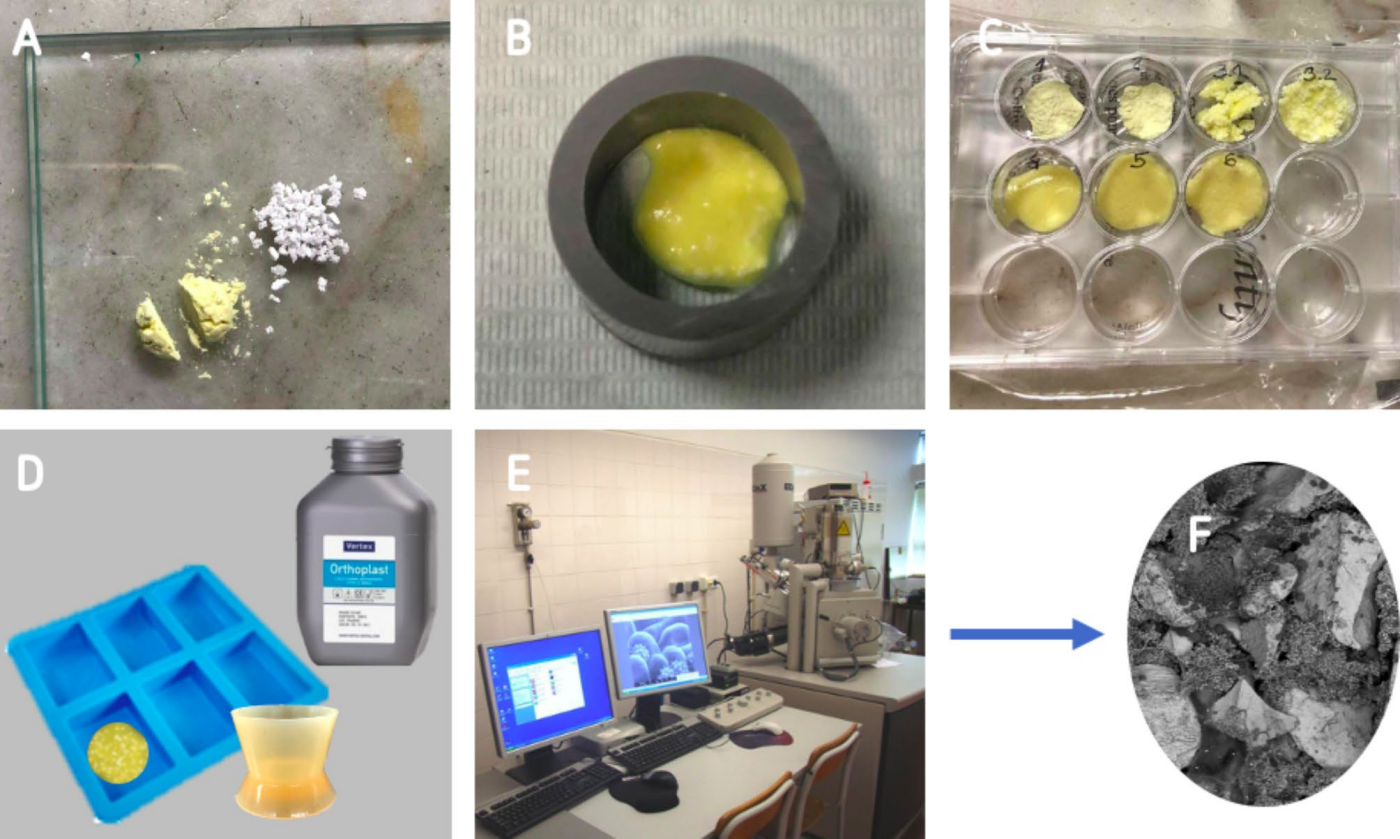
Preparation of specimens. (**A**) Mixture of the materials, (**B**) agglutination and setting time, and (**C**) storage in sterile well-plates. (**D**) Embedment of materials were autopolymerizing polyether-modified resin for (**E**) SEM apparatus and (**F**) image

In this study, 6 groups of specimens were prepared as follow: two control groups with DBBM or HDBG granules without mixing with alginate-based hydrogel; four test groups with DBBM and HDBG granules mixed with alginate-based hydrogel into a putty consistency mixture at different proportions (Fig. 1). For test groups, DBBM or HDBG was mixed with alginate-based hydrogel (Orthoprint™, Zhermack, Germany) at bone graft/hydrogel ratio of 1:1 or 1:3 vol/vol under the sterile condition at room temperature. The volume of the materials was carefully controlled using a sterile spoon which was levelled using a clinical spatula. The materials were mixed for 30 s and placed in a moulding container over a period of 2 min. After the setting time, bone substitutes/hydrogel assemblies were placed in sterile polyester well-plates. Then, bone substitutes/hydrogel assemblies were embedded in autopolymerizing polyether-modified resin (Technovit 400™; Kulzer GmbH, Germany) for later analyses by scanning electron microscopy (SEM), as illustrated in Fig. 2.

### Scanning electron microscopy

Groups of DBBM or HDBG granules embedded in autopolymerizing polyether modified resin were cross-sectioned using a high-precision cutting machine. Surfaces were wet ground down to 2400 Mesh using SiC abrasive papers. Surfaces were ultrasonically cleaned in isopropyl alcohol for 10 min and then in distilled water for 10 min. Surfaces of the cross-sectioned specimens were sputter coated with a AgPd thin layer for scanning electron microscopy (SEM) analyses using a SEM unit (JSM-6010 LV™, JEOL, Japan) coupled to energy dispersive spectroscopy (EDS) (Fig. 2E). Also, DBBM or HDBG granules without mixture with alginate-based hydrogel were analyzed. The dimensions of pores and the distribution of particulate material were evaluated at magnification ranging from x40 up to x20000 under secondary electrons (SE) and backscattered electrons (BSE). Adobe Photoshop™ software program (Adobe Systems Software, Ireland) was used to analyze black and white images, with the black regions representing the pores and the white regions representing the bulk material. Image J™ software program (National Institutes of Health, USA) was used to quantify the dimensions of the granules and pores on the SEM images. A number of three micrographs were acquired at three different magnification, namely x40, x1000, x20000, for each specimen (*n* = 27). The cross-sectioning of specimens and SEM analyses were performed at the CMEMS laboratories at the University of Minho (Portugal).

### Statistical analyses

Results were statistically analyzed by normality test Shapiro-Wilk and two-way ANOVA to determine statistical differences in the size of granules and distance among granules for both biomaterials. The t student test was used to compare values of size, pores, and distance. A probability value < 0.05 was considered significant. The power analysis was performed by t student test or ANOVA to determine the number of samples for each group (n), and to reveal a test power of 100% in the present study. Data on size of granules and distance among granules were harvested directly in Microsoft Office Excel 2016 (Microsoft Corporation, Redmond, WA, USA) and the statistical analyses were carried out using Origin Lab statistical software program (Origin Lab, Northampton, MA, USA).

## Results

**Fig. 3:**
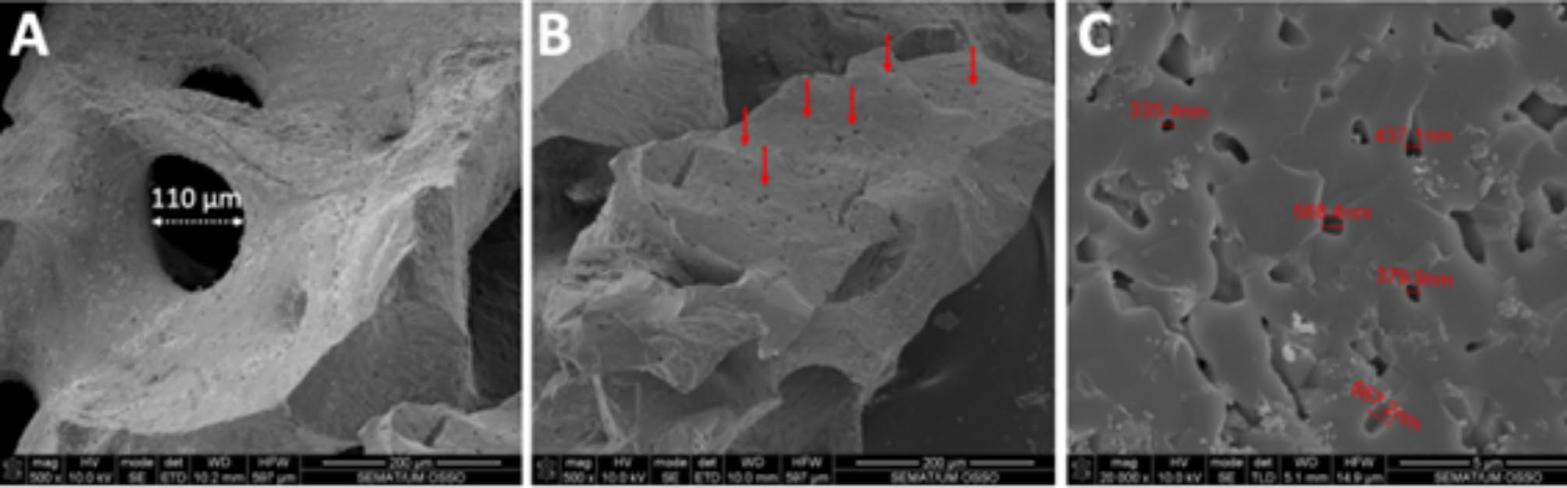
SEM images of DBBM granules at (**A**,**B**) x500 and (**C**) x20000 magnification. Red arrows indicate micro-scale pores present on the surface of DBBM granules

Scanning electron microscopy (SEM) images of DBBM granules are shown in Fig. 3.

**Fig. 4:**
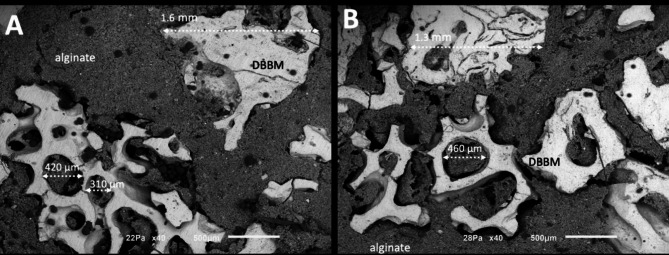
SEM images of DBBM granules into a mixture with alginate-based hydrogel at the DBBM/alginate ratio of 1:3 (**A**) and 1:1 (**B**) vol/vol. SEM images at 40x magnification

As seen in Fig. 3A and B, SEM images at x500 magnification showed the morphological aspects of the DBBM granules without alginate-based hydrogel. DBBM granules showed irregular morphological aspects with macro-scale pores ranging between 50 and 460 μm in diameter (Figs. 3A and 4). Micro-scale pores were inspected on the surface of DBBM granules as seen in Fig. 3C. Micro-scale pores ranged from approximately 330 up to 670 nm in diameter (Figs. 3C and 4).

SEM images of different concentrations of DBBM granules mixture with alginate-based hydrogel into putty mixture are shown in Fig. 4. SEM images revealed a mean size of DBBM granules ranging from 350 up to 1600 μm as seen in Fig. 4. Statistical data on the size of granules is given in Table 1. No significant differences for the size of granules were found between DBBM and HDBG granules (*p* > 0.05).

**Table 1:** Analysis of variance of the size of granules of deproteinized bovine bone mineral (DBBM) or human dentin-derived bone graft (HDBG). Each measurement corresponds to an individual sample. *d.f.*, degrees of freedom; F, Fisher–Snedecor distribution

Variation	Square sum	d.f.	Square average	(F)		*p*-value		
**DBBM**								
Between Groups	62.005	3	62.0054	226.470		> 0.05		
Within Groups	13.988	243	24.287					
Total	75.993	27						
**HDBG**								
Between Groups	78.650	3	78.650	166.078		> 0.05		
Within Groups	15.726	24	2.6134					
Total	94.376	27						

**Fig. 5:**
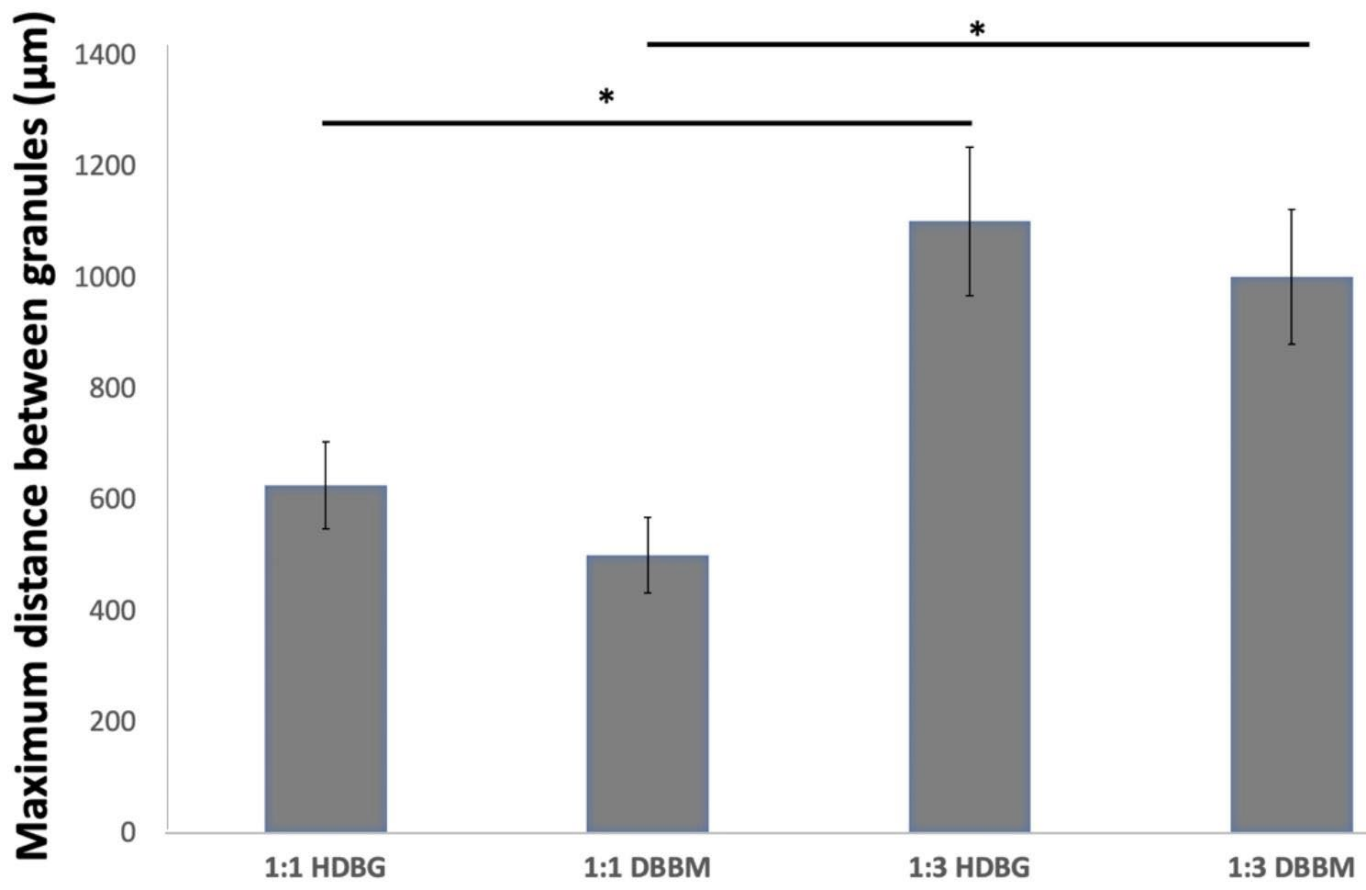
Mean values and standard deviation recorded for the distance among HDBG or DBBM granules. Horizontal bars comparing groups and * indicate statistically significant differences (*p* < 0.05)

The highest distance among DBBM particles was recorded around 1000 μm for groups mixed at 1:3 ratio (Fig. 4A) when compared with the maximum distance of 500 μm among particles mixed at a ratio of 1:1 (Fig. 4B) (*p* < 0.05). The lowest distance among DBBM particles was recorded at 125 μm for particles mixed at 1:1 ratio when compared with particles mixed at 1:3 ratio that revealed a minimum distance at 250 μm (*p* < 0.05). In Fig. 4B, the maximum size of the DBBM macro-pores can be noted at around 460 μm. The maximum distance among DBBM granules is shown in Fig. 5 (*p* < 0.05). Significant statistic differences were recorded between the content of DBBM as seen in Fig. 5; Table 2.

**Table 2:** Analysis of variance of the distance among granules of deproteinized bovine bone mineral (DBBM) or human dentin-derived bone graft (HDBG). Each measurement corresponds to an individual sample. *d.f.*, degrees of freedom; F, Fisher–Snedecor distribution

Variation	Square sum	d.f.	Square average	(F)	*p*-value
**1:1 ratio of DBBM**					
Between Groups	182.7	1	182.7	116.139	<0.005
Within Groups	13.42	8	1.24		
Total	196.12	9			
**1:1 ratio of HDBG**					
Between Groups	138.4	1	138.4	368.013	<0.005
Within Groups	12.98	8	1.3		
Total	151.38	9			
**1:3 ratio of DBBM**					
Between Groups	1,174	1	1,174	668.013	<0.005
Within Groups	121,2	8	2.1		
Total	1,295,2	9			
**1:3 ratio of HDBG**					
Between Groups	1,019	1	1,019	897.650	<0.005
Within Groups	91.1	8	1.8		
Total	1,110.1	9			

**Fig. 6:**
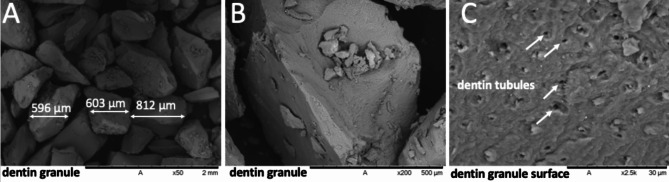
SEM images of HDBG granules at different magnification: (**A**) x50, (**B**) x200, and (**C**) x2500 magnification. (**C**) Dentin tubules can be seen on the surface of dentin granules

SEM images of HDBG granules are shown in Fig. 6.

In Fig. 6A, SEM images at x50 magnification revealed the morphological aspects of the HDBG granules without alginate-based hydrogels. HDBG granules showed irregular morphological aspects with different dimensions ranging from 375 up to 1500 μm after the milling procedure. Statistical data on the size of granules is given in Table 1. Clusters of small fragments were detected on the dentin granule surface at x200 magnification (Fig. 6B). Dentin tubules were also detected on the dentin granule surface at x2500 magnification with pores’ size of around 1–3 μm (Fig. 5C).

**Fig. 7:**
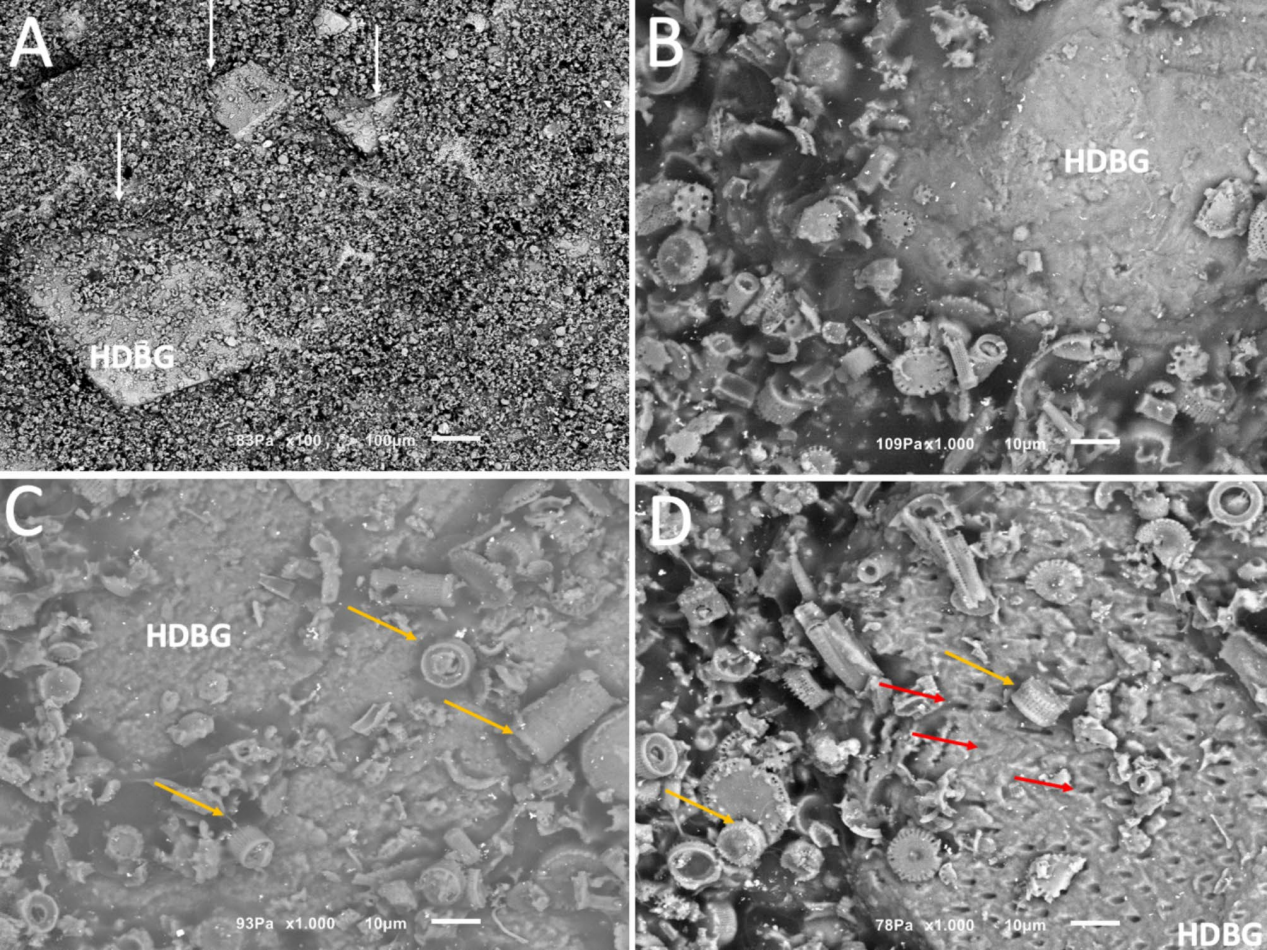
SEM images of HDBG granules (white arrows) into a mixture with alginate-based hydrogel at DBBM/alginate ratio of 1:1 vol/vol. Diatoms (yellow arrows) and dentin tubules (red arrows) can be seen on the surface of the HDBG granules. SEM images at (**A**) x100 and (**B, C, D**) x1000 magnification

**Fig. 8:**
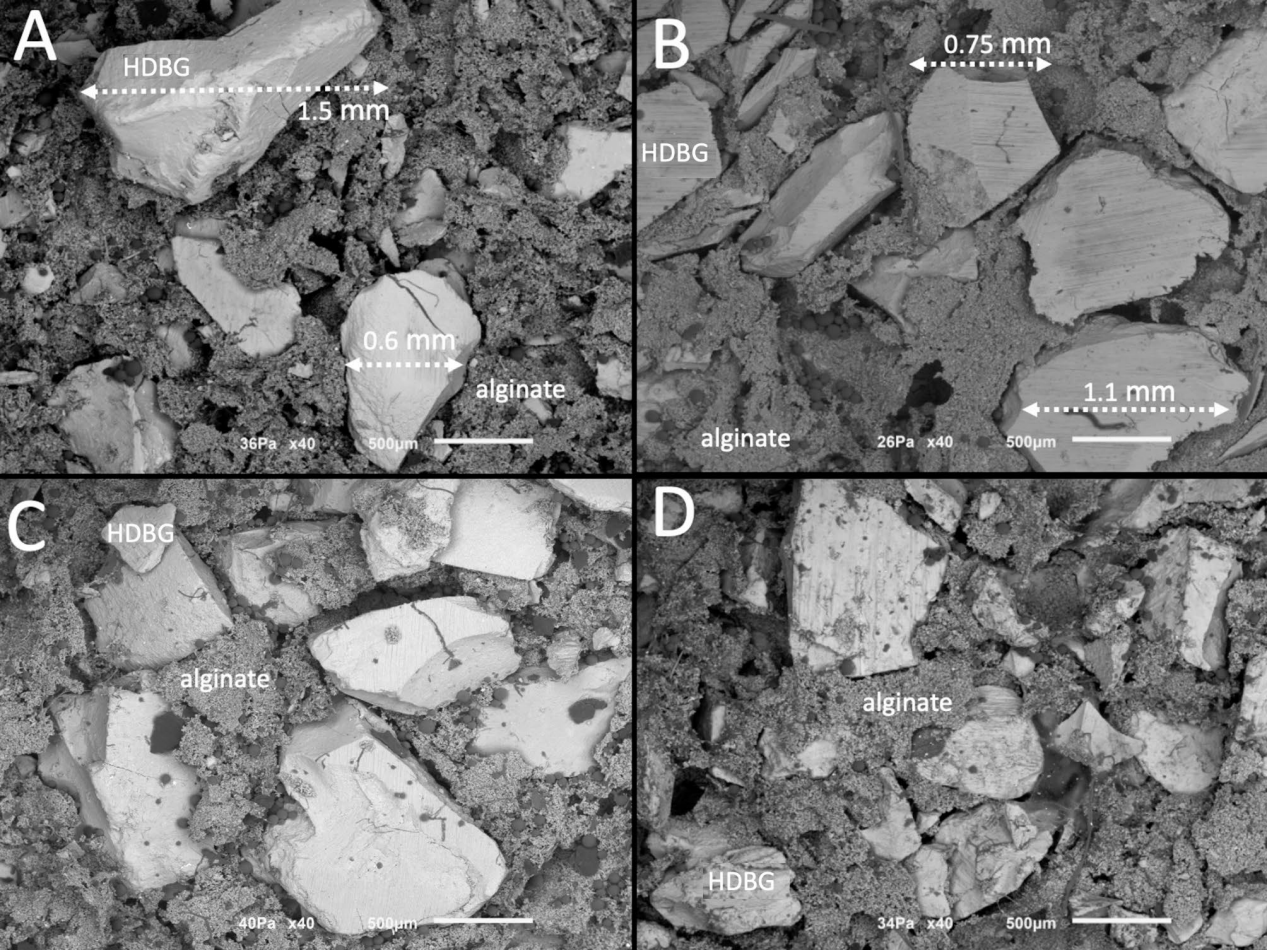
SEM images of HDBG granules mixture with alginate-based hydrogel at the proportions DBBM/alginate (**A,B**) 1:3 and (**C,D**) 1:1 vol/vol. SEM images at 40x magnification

SEM images of different concentrations of HDBG granules mixed with alginate-based hydrogel into putty consistency mixture are shown in Figs. 7 and 8. In Fig. 7, HDBG granules (white arrows) mixed with alginate-based hydrogel were detected by SEM images at x100 magnification. At high magnification, HDBG granules were noticed surrounded by numerous unicellular cells, named diatoms (yellow arrows), which composed the alginate-based hydrogel. Dentin tubules were also detected on the surface of the HDBG granules.

In Fig. 8, SEM images validate a size of HDBG granules ranging from 375 up to 1500 μm. The highest distance among particles was recorded around 1100 μm for groups mixed at 1:3 ratio (Fig. 8A) when compared with the maximum distance of 625 μm among particles mixed at 1:1 ratio (Fig. 8B) (*p* < 0.05). The lowest distance among particles was recorded at 125 μm for both mixture proportions (*p* < 0.05). The maximum distance among HDBG granules is shown in Fig. 5 (*p* < 0.05). Significant statistic differences were recorded for the distance among HDBG granules as seen in Fig. 5; Table 2.

## Discussion

The morphology and distribution of deproteinized bovine bone mineral (DBBM) and human dentin-derived bone graft (HDBG) into a putty consistency mixture were analyzed under scanning electron microscopy. Data on the size of granules revealed no statistical differences among the groups of materials. Nevertheless, the results of the present study support the hypothesis that the distance between DBBM or HDBG granules vary into a putty mixture regarding the proportion of granules. In fact, a high content of bioactive ceramic granules into a putty consistency mixture with hydrogels decrease the space among granules providing space for cell adhesion and formation of vessels on a bone defect repairing.

In the present study, DBBM and HDBG particles (granules) showed dimensions ranging from 350 up to 1600 μm although smaller granules could be found within the powders. The variation in the dimensions of the granules is adequate for clinical handling since small granules are entrapped among larger granules. Then, the compaction of the granules can promote a high density of bioactive ceramics and an increased number of nucleation area for osteogenic cells into the bone defect. The dimensions of the granules are also in line with those recommended by other studies [2, 4–7, 19, 9]. Several studies found that block-based bone substitutes placed as a putty consistency mixture provide higher volume stability when compared to granules. However, block-based bone substitutes showed less new bone ingrowth in comparison with the particulate materials [2, 4–7, 19, 9]. Such difference in the percentage of new bone can be partially explained due to differences in the granules aspects and contact area for blood vessel infiltration (angiogenesis) and new bone ingrowth [5, 20, 21]. DBBM granules could be more densely filled into the same size defect when compared to DBBM blocks regarding volume and surface area of bioactive inorganic material [4, 6, 10].

Another study reported small inorganic bovine bone granules < 450 μm are adequate for bone formation when compared with wider granules ranging from 450 up to 749 μm or 750 up to 1000 μm [10]. Indeed, small granules provide higher surface area for interaction with the surrounding media which includes proteins, osteogenic cells, and blood products [6, 10]. A previous study demonstrate that small particle fragments attract more mono and multinucleated giant cells when compared to regular DBBM granules [4]. Thus, small Hap fragments (< 20 μm) strongly induce a transient inflammatory response. However, a chronic inflammation causes osteolysis and suppresses bone formation, compromising the tissue healing. Another study reported that both small (0.25-1 mm) and large (1–2 mm) granules composed of DBBM were equally effective in bone formation [22]. Thus, the migration of cells and formation of blood vessels prior to bone formation also depends on the distance of the granules or else on the size of macro-scale pores [7, 23] as shown in the present study (Figs. 3 and 4).

Regarding the proportion of granules into a putty mixture, a decrease of DBBM or HDBG provided a large distance among particles leading to a contact osteogenesis. In this study, the distance among DBBM particles at a 1:3 ratio (DBBM/hydrogel) ranged from 250 up to 1000 μm while a 1:1 ratio (DBBM/hydrogel) provide distances at a range from 125 up to 500 μm. The distance among HDBG particles in the 1:3 ratio (HDBG/hydrogel) ranged from 125 up to 1100 μm while a 1:1 ratio (HDBG/hydrogel) provide distances at a range from 125 up to 625 μm. A large distance among granules provides a high content of the hydrogel which is rapidly absorbed leading to a high shrinkage of the tissue over the bone healing process. A short distance among particles below 1 mm is proper for the performance of osteogenic cells and the pathways of bone healing by contact osteogenesis. According to some studies, spaces among granules of around 300 to 500 μm promoted a higher bone formation when compared with spaces of 50 to 100 μm among granules [6, 21]. Spaces among granules must be wider than 100 μm for adequate formation of vessels and new bone [21]. Taking into account the literature data, an increase in the bone substitute content in a putty mixture provide proper spaces among granules [6, 21]. Thus, an increase in DBBM or HDBG in the mixture also maintain the bone volume for further oral rehabilitation procedures.

As seen in Fig. 3, the size of macro-scale pores recorded for DBBM ranged from 50 up 460 μm in diameter that is proper for the formation of blood vessels while the micro-scale pores ranged between 0.3 and 0.7 μm. The macro- and micro-scale pores for retention of the hydrogel over the handling procedure can provide a drug delivery system when placed into the bone defect. The pores are retaining spots for adhesion of osteogenic cell as well as the adsorption and gradually delivery of molecules, minerals, and proteins. Studies have shown that osteogenic cells with size ranging between 10 and 50 μm prefer macro-scale pores at around 200 μm leading to an enhanced bone formation [6, 21]. In osteogenesis, osteoblasts and pre-osteoblasts present in the material stimulate an ossification reaction. Therefore, porous materials work as a scaffold that allows cell adhesion and formation of blood vessels [4–6, 19]. Several studies have shown that particle size, porosity, and size of the pores strongly influences osteoconduction and new bone densification as well as the migration of osteoblasts and mesenchymal cells into the graft material [6, 7, 12]. Human dentin-derived graft can be considered as a potential material for bone healing regarding the calcium- and phosphate-based composition. After grinding, the amount of HDBG granules is suitable for filling a bone defect with the same volume from the source site. The porosity of the HDBG granules is around 44.5% that can be beneficial for adsorption of blood products and for the resorption process by macrophages. Dentin-derived matrix with a space between granules of 200 μm was effective in bone formation, suggesting materials with a small particle size could reasonably be used for bone grafting. Also, bone substitutes with lower porosity showed similar or higher bone formation when compared with autogenous bone [6, 7, 12]. Macro-scale pores also allow the infiltration of macrophages to combat bacteria and stimulate the infiltration of blood cells and osteogenic cells. Pro-inflammatory cytokines are also secreted at higher levels in macro-scale pores, that can trigger bone healing. On the other hand, smaller pores decrease the cell aggregation and proliferation although micro-scale pores are associated with a hypoxic state that stimulates endothelial cell proliferation [23]. Micro-scale pores are retentive spots for the retention and gradually delivery of molecules, minerals, and proteins such as BMP and growth factors [23]. Additionally, micro-scale pores are retentive regions for the adhesion and spreading osteogenic cells.

Regarding the HDBG assessed in the present study, granules were found ranging from 375 up to 1500 μm (Fig. 6). Clusters of small fragments were detected on the surface of dentin granules at x200 magnification (Fig. 6). Smalls fragments affect the magnitude of inflammatory response that is also dependent on the bioactivity and resorption rate of the fragments. In another study, granules of partially demineralized dentin matrix (PDDM) or completely demineralized dentin matrix (CDDM) of larger size (800–1200 μm) showed enhanced bone formation when compared with granules at smaller size (180–212 μm or 425–600 μm [16]. However, PDDM or CDDM granules were reabsorbed even before bone formation began. Adequate rate of resorption and bone formation were recorded for small granules of highly mineralized bone substitutes such as DBBM or HDBG. Indeed, the balance between matrix resorption and bone formation is vital for enhanced bone healing [16].

As seen in Figs. 6 and 7, HDBG granules showed only the presence of micro-scale pores as a result from the dentin tubules. Micro-scale pores derived from dentinal tubules of 1–3 μm are too small for cell infiltration and growth although osteogenic cells used the pores for adhesion and spreading over the particles’ surfaces [6, 8, 15]. Dentinal tubules of HDBG granules are enlarged after the deproteinizing procedures and can serve as channels to exchanging of proteins and ions from blood fluids which are essential for osteoblast growth and differentiation [8]. Dentinal tubules in dentin can be enlarged by varying the demineralization process, resulting in increased porosity from 3 up to 20% [23–25]. In an experimental study in rats, demineralized deciduous tooth powder were prepared by applying different demineralization time to improve the porous structure and surface area for cell adhesion [26]. Bone healing was detected in grafted sites and the deciduous teeth revealed structural and physicochemical characteristics suitable for grafting with appropriate demineralization. Bone healing was observed to have successfully occurred in DDTP-grafted sites. However, excessive demineralization of dentin can damage the dentin structure and lead to resorption, resulting in insufficient bone substitute. Also, an inadequate demineralization process produces a bone substitute with poor osteogenic properties [23–25].

Micro-scale pores are still important for the pathways of adsorption and delivery of molecules, minerals, and proteins including growth factors. In osteoinduction, chemotactic materials capable of attracting other cells such as BMP can induce migration and differentiation of mesenchymal cells into chondroblasts or osteoblasts [16]. Limited porous granules work as an impermeable wall that prevents cell infiltration and vascularization and therefore the formation of blood vessels could occur only among the granules since the minimum distance was recorded at 250 μm [7, 15]. A previous study reported that macro-scale pores shaped at 1 mm in diameter in root dentin matrix blocks enhanced active bone growth [15]. The increase in the pores’ size and overall macro-scale porosity induced the osteoconductive of the scaffold leading to adequate space for the fixation, differentiation, and proliferation of osteoblasts [8, 27].

Based on this in vitro model, further steps should be performed considering the distribution and size of granules into hydrogel mixtures. The variation in the proportion of bioactive synthetic and natural ceramic granules is a major factor for future studies. Also, clinical guidelines could be recommended concerning a balance on the density of bioactive ceramics and content of hydrogels. Several bioactive materials can be used as hydrogel such as collagen, chitosan, hyaluronic acid, and fibrin. For instance, a coupling between collagen and alginate can improve the cell adhesion in the hydrogel mixture. Thus, the delivery and adsorption of proteins and ions should be assessed regarding the chemical composition, content, and structure of the hydrogels and mineral granules. Then, biological assays with different cell lines followed by animal studies can validate the effects of different content and size of dentin-derived granules. Further studies are required to understand the pathways of chemical interaction between proteins, osteogenic cells, and porous dentin-derived granules to provide guidelines for clinical use in bone healing procedures.

## Conclusions

From this study, the concluding conclusions could be drawn:

- Deproteinized bovine bone mineral (DBBM) and human dentin-derived bone graft (HDBG) can be produced with particle size at similar dimensions ranging from 350 up to 1600 μm, revealing that some particles (granules) can reach small or higher dimensions considering the processing parameters;
- The size of pores of DBBM granules were found at macro-scale (~ 50–460 μm) and at micro-scale dimensions (~ 0.3–0.7 μm). On HDBG, the pores were found only at micro-scale dimensions (1–3 μm) that was represented by dentin tubules. Micro-scale pores are important for adsorption of proteins and adhesion followed by spreading of osteogenic cells while macro-scale pores are crucial for angiogenesis and bone formation;
- The distance of DBBM or HDBG granules was higher (around 1 mm) for mixtures containing a high content of alginate-based hydrogel.
- The decrease of DBBM or HDBG granules into a putty mixture provide a high volume of hydrogels that would be rapidly resorbed in the bone healing process. In a clinical application, the decrease of the bioactive ceramic granules can negatively affect the mechanical stability of the bone defects over the healing process. Also, the lower content of bone substitute can induce the tissue shrinkage during the bone healing process.

## Data Availability

All data generated or analyzed during this study are included in this published article. The datasets used and/or analysed during the current study available from the corresponding author on reasonable request.
